# Novel Variant and Known Mutation in 23S rRNA Gene of *Mycoplasma pneumoniae*, Northern Vietnam, 2023

**DOI:** 10.3201/eid3005.231632

**Published:** 2024-05

**Authors:** Dinh-Dung Nguyen, Nhan Thi Ho, Lynn G. Dover, Anh Hang Mai Vo, Ha Thi Thanh Ly, Phuong Mai Doan, Hang Thi Nguyen, Nguyen Thi Thao Luu, An Nhat Pham, Huyen Thi Thanh Tran

**Affiliations:** Author affiliations: Vinmec Healthcare System, Hanoi, Vietnam (D.-D. Nguyen, N.T. Ho, A.H.M. Vo, H.T.T. Ly, P.M. Doan, H.T. Nguyen, N.T.T. Luu, A.N. Pham, H.T.T. Tran); Northumbria University, Newcastle upon Tyne, UK (L.G. Dover)

**Keywords:** Mycoplasma pneumoniae, macrolide-resistant mutation, C2353T, A2063G, bacteria, community-acquired pneumonia, Vietnam

## Abstract

During a 2023 outbreak of *Mycoplasma pneumoniae*–associated community-acquired pneumonia among children in northern Vietnam, we analyzed *M. pneumoniae* isolated from nasopharyngeal samples. In almost half (6 of 13) of samples tested, we found known A2063G mutations (macrolide resistance) and a novel C2353T variant on the 23S rRNA gene.

*Mycoplasma pneumoniae* is a common etiologic agent of community-acquired pneumonia (CAP) among children. Although *M. pneumoniae* infection often causes a mild and self-limiting disease, pneumonia develops in ≈10%–20% of pediatric patients (*1*). First-line therapies for *M. pneumoniae* infection are based on macrolides, a group of antimicrobial drugs widely used in outpatient settings because of their high oral bioavailability. However, overuse and indiscriminate use of macrolides have contributed to the emergence of macrolide-resistant *M. pneumoniae* (MRMP). Point mutations in the V region of the *M. pneumoniae* 23S rRNA gene have been associated with macrolide resistance (*2*). In recent years, prevalence of MRMP has increased and is very high in Asia (13.6%–100%) (*2*–*4*). During spring/summer 2023, hundreds of children with CAP were admitted daily to each of the major hospitals in Hanoi, Vietnam. *M. pneumoniae* has emerged as the major pathogen detected in approximately one third of patients with CAP (5). We analyzed the mutations in the 23S rRNA gene of *M. pneumoniae* isolated from nasopharyngeal samples of pediatric CAP patients during the 2023 outbreak in Vinmec Times City Hospital, Hanoi.

During May 1–July 31, 2024, the real-time PCR Allplex Respiratory Panel 4 detected *M. pneumoniae* in 411 (26.1%) of 1,578 nasopharyngeal samples from children with suspected CAP. Among *M. pneumoniae*–positive samples with a cycle threshold <30, we randomly selected 13 samples from 13 patients for gene sequencing. We amplified the DNA sequence of the 748-bp region (nt 1963–2710) of the 23S rRNA gene containing all known MRMP mutations by using MRMP-F1 (5′-CGTCCCGCTTGAATGGTGTA-3′) and MRMP-R1 (5′-GGCGCTACAACTGGAGCATA-3′). We sequenced the amplicons according to the Sanger sequencing method by using a BigDye Terminator v3.1 Cycle Sequencing Kit and Applied Biosystems 3500 Dx Genetic Analyzer instrument (both Thermo Fisher Scientific, https://www.thermofisher.com). We assembled the generated sequence data and analyzed them for variations by comparing with the reference *M. pneumoniae* strain M129 23S ribosomal RNA gene (GenBank accession no. NR_077056.1), using BLAST (http://blast.ncbi.nlm.nih.gov). We used ClustalW to perform multiple alignments (*6*). Subsequently, we constructed the phylogenetic tree according to the maximum-likelihood method with bootstrap analysis (n = 500) by using MEGA11 software (https://www.megasoftware.net). The 2-dimensional secondary structure of the 23S rRNA gene was predicted by the R2DT tool (RNAcentral) according to an SA_LSU_3D template provided by RiboVision (*7*).

Of the 13 samples, 6 (46.2%) showed single-nucleotide variation from the type strain sequence in the V region of the 23S rRNA gene. A known A2063G mutation was detected in 4 samples, and a novel variant C2353T was found in 2 samples (Figure, panel A).

**Figure:**
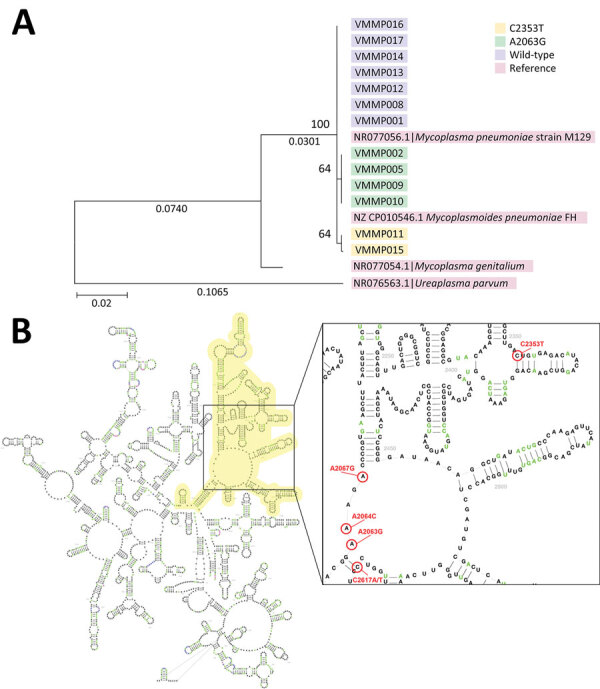
Phylogenetic tree and location of mutations for *Mycoplasma pneumoniae* strains identified in pediatric patients hospitalized with community-acquired pneumonia, Hanoi, Vietnam, spring/summer 2023. A) Maximum-likelihood phylogenetic analysis of the domain V region of the 23S rRNA gene. B) Predicted RNA secondary structure of 23S rRNA gene constructed with the description of known mutations (A2063G/C/T, A2064G, A2067G, C2617G) and novel variant (C2353T). Yellow highlights indicate the domain V region of 23S rRNA. Scale bar indicates base substitutions per site.

The known MRMP mutation A2063G is the most prevalent mutation reported to date compared with other infrequent mutations (e.g., A2063T/C, A2064G, A2067G, A1290G, and C2617A) (*2*,*8*). Mutations at site 2063 are also associated with a high level of macrolide resistance (*9*,*10*). The National Institutes of Health databases showed no recorded evidence for the sequences containing the C2353T variant observed in our study (Figure, panel B). We hypothesize that under selection pressure during CAP treatment with macrolides, C2353T mutants have emerged with macrolide resistance. Previous reports have shown that different mutations can lead to different levels of macrolide affinity as well as MIC elevation (*8*). Demonstration of MRMP by culture and MIC is not regularly done in clinical practice; thus, rapid detection of MRMP mutation may provide useful information for guiding antimicrobial drug therapy.

Clinical nonresponse to initial macrolide treatment was experienced by 3 (50%) of the 6 patients with the novel or known mutation and 2 (28.6%) of the 7 without (Table; Appendix Table). Other respiratory bacteria were co-detected in approximately two thirds of patients in both groups, which might also affect clinical characteristics.

**Table:** Summary of patient characteristics in study of novel variant and known mutation in 23S rRNA gene of *Mycoplasma pneumoniae*, northern Vietnam, 2023*

Characteristic	No mutation, n = 7	23S rRNA gene mutation, n = 6	Total, n = 13
Mutation type			
A2063G	0	4 (66.7)	4 (30.8)
C2353T	0	2 (33.3)	2 (15.4)
PCR panel of 7 respiratory bacteria	7 (100.0)	6 (100.0)	13 (100.0)
Positive with *Mycoplasma pneumonia*	7 (100.0)	6 (100.0)	13 (100.0)
Positive with *Hemophilus influenzae*	2 (28.6)	2 (33.3)	4 (30.8)
Positive with *Streptococcus pneumoniae*	4 (57.1)	3 (50.0)	7 (53.8)
Positive with other bacteria	0	0	0
Nasopharyngeal culture			
Negative	3 (42.9)	2 (33.3)	5 (38.5)
Positive	2 (28.6)	0	2 (15.4)
Not done	2 (28.6)	4 (66.7)	6 (46.2)
Culture result			
* H. influenzae*	1 (50.0)	0	1 (50.0)
* S. pyogenes*	1 (50.0)	0	1 (50.0)
Co-detection of other bacteria by PCR panel	4 (57.1)	4 (66.7)	8 (61.5)
Co-detection of other bacteria by PCR panel or culture	5 (71.4)	4 (66.7)	9 (69.2)
*Mycoplasma* IgM test done	2 (28.6)	2 (33.3)	4 (30.8)
Positive result	1 (50.0)	2 (100.0)	3 (75.0)
Influenza antigen rapid test done	1 (14.3)	2 (33.3)	3 (23.1)
Positive result	0	0	0
Patient sex			
M	4 (57.1)	2 (33.3)	6 (46.2)
F	3 (42.9)	4 (66.7)	7 (53.8)
Patient age, y (max median, min median)	4.000 (1, 7)	5 (2, 13)	4 (1, 13)
Hospitalized	5 (71.4)	3 (50.0)	8 (61.5)
Day of illness at hospital visit and nasopharyngeal sample aspiration, median (min, max)	5 (4, 7)	4 (3, 5)	5 (3, 7)
Days with fever at hospital visit (min median, max median)	5 (2, 5)	4 (3, 5)	4 (2, 5)
Cough	7 (100.0)	6 (100.0)	13 (100.0)
Shortness of breath	0	1 (16.7)	1 (7.7)
Wheezing	0	1 (16.7)	1 (7.7)
Chest pain	1 (14.3)	0	1 (7.7)
Pulmonary rale	4 (57.1)	4 (66.7)	8 (61.5)
Lung lesion on chest radiograph	7 (100.0)	6 (100.0)	13 (100.0)
Oxygen supplement	0	1 (16.7)	1 (7.7)
Treatment before hospital visit	2 (28.6)	4 (66.7)	6 (46.2)
Antibiotic treatment before hospital visit			
Amoxicillin and clavulanic acid	1 (14.3)	1 (16.7)	2 (15.4)
Azithromycin	1 (14.3)	0	1 (7.7)
Clarithromycin	0	1 (16.7)	1 (7.7)
Antibiotic treatment duration before hospital visit, d (min median, max median)	3.5 (3,4)	4 (3,6)	4 (3,6)
Macrolide initiated at the beginning of hospital treatment	6 (85.7)	6 (100.0)	12 (92.3)
Azithromycin	4 (57.1)	5 (83.3)	9 (69.2)
Clarithromycin	2 (28.6)	1 (16.7)	3 (23.1)
Other antibiotic in combination at the beginning of hospital treatment			
Amoxicillin/clavulanic acid	4 (57.1)	4 (66.7)	8 (61.5)
Cefdinir	0	1 (16.7)	1 (7.7)
Ceftriaxone	2 (28.6)	1 (16.7)	3 (23.1)
Switch to clarithromycin	0	2 (66.7)	2 (33.3)
After 5 d	0	1 (50.0)	1 (50.0)
After 6 d	0	1 (50.0)	1 (50.0)
Other initial antibiotic			
Amoxicillin/clavulanic acid	1 (14.3)	0	1 (7.7)
Macrolide used after other initial antibiotic	1 (14.3)	0	1 (7.7)
Azithromycin (1 d after other initial antibiotic)	1 (14.3)	0	1 (7.7)
Clinical response to initial antibiotic treatment	5 (71.4)	3 (50)	8 (61.5)
Clinical evaluation after how many days of initial macrolide treatment median (min, max)	2.5 (2, 3)	3 (3, 3)	3 (2, 3)
Clinical nonresponse to initial antibiotic treatment			
Persistent fever	2 (28.6)	1 (16.7)	3 (23.1)
Persistent or worse chest radiograph	0	2 (28.6)	2 (15.4)
Persistent respiratory distress	0	1 (16.7)	1 (7.7)
New respiratory distress	2 (28.6)	0	2 (15.4)
Alternative antibiotic			
Clarithromycin	0	2 (33.3)	2 (15.4)
Levofloxacin	2 (28.6)	1 (16.7)	3 (23.1)
Response to alternative antibiotics	2 (28.6)	3 (50.0)	5 (38.5)
Treatment result			
Discharged with no complication	5 (71.4)	3 (50.0)	8 (61.5)
Recovered with outpatient treatment	2 (28.6)	3 (50.0)	5 (38.5)
*Data are no. (%) unless otherwise indicated.			

In summary, we detected the novel C2353T variant and known A2063G mutations in the 23S rRNA gene in nearly half of the pediatric patients with *M. pneumoniae*–associated CAP in Vinmec Times City Hospital during the 2023 outbreak in northern Vietnam. Our findings are consistent with those of other studies regarding the rising prevalence of MRMP in Southeast Asia. Our study findings may indicate circulation of different MRMP variants in Vietnam or emergence of new MRMP variants during the recent *M. pneumoniae*-associated CAP outbreak among children.

Appendix Additional information for study of novel variant and known mutation in 23S rRNA gene of *Mycoplasma pneumoniae*, northern Vietnam, 2023.
