# Supplementary material for: Novel Variant and Known Mutation in 23S rRNA Gene of Mycoplasma pneumoniae, Northern Vietnam, 2023
Source: Emerg Infect Dis. 2024 May;30(5):1034–6. doi: 10.3201/eid3005.231632 (PMC11060462; doi:10.3201/eid3005.231632)
Supplement: Appendix — Additional information for study of novel variant and known mutation in 23S rRNA gene of Mycoplasma pneumoniae, northern Vietnam, 2023. [file 23-1632-Techapp-s1.pdf]

Article DOI: https://doi.org/10.3201/eid3005.231632

EID cannot ensure accessibility for supplementary materials supplied by authors. Readers who have difficulty accessing supplementary content should contact the authors for assistance.

Novel Variant and Known Mutation in 23S rRNA Gene of *Mycoplasma pneumoniae*, Northern Vietnam, 2023

Appendix

Appendix Table. Characteristics of each of all patients

Patient	1	2	3	4	5	6	7	8	9	10	11	12	13
Mutation	Yes	Yes	Yes	Yes	Yes	Yes	No	No	No	No	No	No	No
Mutation type	A2063G	A2063G	A2063G	A2063G	C2353T	C2353T	Non-detected	Non-detected	Non-detected	Non-detected	Non-detected	Non-detected	Non-detected
Nasopharyngeal culture	Negative	Not done	Not done	Not done	Negative	Not done	Not done	Positive	Negative	Not done	Positive	Negative	Negative
- Positive Bacteria	NA	NA	NA	NA	NA	NA	NA	H. influenzae	NA	NA	S. pyogenes	NA	NA
PCR panel of 7 respiratory bacteria													
- Positive with M. pneumoniae	Yes	Yes	Yes	Yes	Yes	Yes	Yes	Yes	Yes	Yes	Yes	Yes	Yes
- Positive with H. influenzae	No	No	Yes	No	Yes	No	No	Yes	No	No	No	No	Yes
- Positive with S. pneumoniae	No	No	No	Yes	Yes	Yes	No	Yes	No	Yes	No	Yes	Yes
- Positive with other bacteria	No	No	No	No	No	No	No	No	No	No	No	No	No
Co_detection of bacteria by PCR panel	No	No	Yes	Yes	Yes	Yes	No	Yes	No	Yes	No	Yes	Yes
Co_detection of bacteria by PCR or culture	No	No	Yes	Yes	Yes	Yes	No	Yes	No	Yes	Yes	Yes	Yes
Mycoplasma IgM	Positive	NOT DONE	NOT DONE	Positive	NOT DONE	NOT DONE	NOT DONE	NOT DONE	Negative	NOT DONE	NOT DONE	NOT DONE	Positive
Flu rapid test	NOT DONE	NOT DONE	NOT DONE	NOT DONE	Negative	Negative	NOT DONE	NOT DONE	NOT DONE	NOT DONE	Negative	NOT DONE	NOT DONE
Gender	Female	Female	Male	Female	Male	Female	Male	Female	Female	Female	Male	Male	Male
Age (year)	4	6	4	2	13	6	1	1	4	7	7	4	3

Patient	1	2	3	4	5	6	7	8	9	10	11	12	13
Hospitalized	Yes	No	No	Yes	Yes	No	No	Yes	Yes	No	Yes	Yes	Yes
Date of hospital visit	2023-05-18	2023-06-28	2023-06-27	2023-05-19	2023-01-07	2023-06-28	2023-06-29	2023-02-07	2023-05-22	2023-07-01	2023-01-07	2023-06-26	2023-02-07
Day of illness at hospital visit and nasopharyngeal sample aspiration	5	3	4	5	3	4	5	4	5	7	4	5	5
Days with fever at hospital visit	5	3	4	5	3	4	5	4	5	2	4	5	5
Cough	Yes	Yes	Yes	Yes	Yes	Yes	Yes	Yes	Yes	Yes	Yes	Yes	Yes
Respiratory distress	No	No	No	Yes	No	No	No	No	No	No	No	No	No
Wheezing	No	No	No	Yes	No	No	No	No	No	No	No	No	No
Chest pain	No	No	No	No	No	No	No	No	No	Yes	No	No	No
Pulmonary rales	Yes	No	No	Yes	Yes	Yes	Yes	No	Yes	No	No	Yes	Yes
Lung lesion in chest X-ray	Yes	Yes	Yes	Yes	Yes	Yes	Yes	Yes	Yes	Yes	Yes	Yes	Yes
Oxygen supplementation	No	No	No	Yes	No	No	No	No	No	No	No	No	No
Treatment before hospital visit	Yes	Yes	Yes	No	No	Yes	No	No	Yes	No	No	No	Yes
- Antibiotic use before hospital visit	Amoxicillin & Clavulanic acid	No	Clarithromycin	NA	NA	No	NA	NA	Azithromicin	NA	NA	NA	Amoxicillin & Clavulanic acid
- Duration of treatment before hospital visit	4	3	6	NA	NA	4	NA	NA	3	NA	NA	NA	4
Macrolide initiated at the beginning of hospital treatment	Yes	Yes	Yes	Yes	Yes	Yes	Yes	No	Yes	Yes	Yes	Yes	Yes
- Macrolide type	Azithromycin	Azithromycin	Clarithromycin	Azithromycin	Azithromycin	Azithromycin	Azithromycin	NA	Clarithromycin	Azithromycin	Clarithromycin	Azithromycin	Azithromycin
Other antibiotic use at the beginning of hospital treatment	Ceftriaxone	Amoxicillin & Clavulanic acid	Cefdinir	Amoxicillin & Clavulanic acid	Amoxicillin & Clavulanic acid	Amoxicillin & Clavulanic acid	NA	Amoxicillin & Clavulanic acid	Ceftriaxone	Amoxicillin & Clavulanic acid	Amoxicillin & Clavulanic acid	Amoxicillin & Clavulanic acid	Ceftriaxone
Switch to Clarithromycin	Yes	No	No	No	Yes	No	No	NA	NA	No	No	No	No
- Switch after how many day	6	NA	NA	NA	5	NA	NA	NA	NA	NA	NA	NA	NA
Macrolide was used after initial antibiotic (day)	NA	NA	NA	NA	NA	NA	NA	1	NA	NA	NA	NA	NA
- Macrolide type	NA	NA	NA	NA	NA	NA	NA	Azithromycin	NA	NA	NA	NA	NA

Patient	1	2	3	4	5	6	7	8	9	10	11	12	13
Clinical response to initial macrolide treatment (after >48h)	No	Yes	Yes	No	No	Yes	Yes	No	No	Yes	Yes	Yes	Yes
- Evaluation after how many days of initial macrolide treatment?	3	3	6	3	3	5	2	3	2	6	3	3	3
Clinical non-response													
- Reason: persistent fever	No	No	No	Yes	No	No	No	Yes	Yes	No	No	No	No
- Reason: persistent or worse chest X-xray	Yes	No	No	No	Yes	No	No	No	No	No	No	No	No
- Reason: persistent respiratory distress	No	No	No	Yes	No	No	No	No	No	No	No	No	No
- Reason: new respiratory distress	No	No	No	No	No	No	No	Yes	Yes	No	No	No	No
Alternative antibiotic use	Clarithromycin	No	No	Levofloxacin	Clarithromycin	No	No	Levofloxacin	Levofloxacin	No	NA	No	No
- Response to alternative antibiotic	Yes	NA	NA	Yes	Yes	NA	NA	Yes	Yes	NA	NA	NA	NA
Treatment results	Discharged with no complication	Recovered with outpatient treatment	Recovered with outpatient treatment	Discharged with no complication	Discharged with no complication	Recovered with outpatient treatment	Recovered with outpatient treatment	Discharged with no complication	Discharged with no complication	Recovered with outpatient treatment	Discharged with no complication	Discharged with no complication	Discharged with no complication
